# Health Related Quality of Life among Patients with Tuberculosis and HIV in Thailand

**DOI:** 10.1371/journal.pone.0029775

**Published:** 2012-01-11

**Authors:** Wanitchaya Kittikraisak, Pritaporn Kingkaew, Yot Teerawattananon, Jomkwan Yothasamut, Supalert Natesuwan, Weerawat Manosuthi, Virasakdi Chongsuvivatwong, Sara J. Whitehead

**Affiliations:** 1 Thailand Ministry of Public Health, U.S. Centers for Disease Control and Prevention Collaboration, Nonthaburi, Thailand; 2 Health Intervention and Technology Assessment Program, Ministry of Public Health, Nonthaburi, Thailand; 3 Chiang Rai Regional Hospital, Chiang Rai, Thailand; 4 Bamrasnaradura Infectious Diseases Institute, Nonthaburi, Thailand; 5 Epidemiology Unit, Prince of Songkla University, Songkla, Thailand; 6 Centers for Disease Control and Prevention, Georgia, United States of America; University of California, San Francisco, United States of America

## Abstract

**Introduction:**

Health utilities of tuberculosis (TB) patients may be diminished by side effects from medication, prolonged treatment duration, physical effects of the disease itself, and social stigma attached to the disease.

**Methods:**

We collected health utility data from Thai patients who were on TB treatment or had been successfully treated for TB for the purpose of economic modeling. Structured questionnaire and EuroQol (EQ-5D) and EuroQol visual analog scale (EQ-VAS) instruments were used as data collection tools. We compared utility of patients with two co-morbidities calculated using multiplicative model (U_CAL_) with the direct measures and fitted Tobit regression models to examine factors predictive of health utility and to assess difference in health utilities of patients in various medical conditions.

**Results:**

Of 222 patients analyzed, 138 (62%) were male; median age at enrollment was 40 years (interquartile range [IQR], 35–47). Median monthly household income was 6,000 Baht (187 US$; IQR, 4,000–15,000 Baht [125–469 US$]). Concordance correlation coefficient between utilities measured using EQ-5D and EQ-VAS (U_EQ-5D_ and U_VAS_, respectively) was 0.6. U_CAL_ for HIV-infected TB patients was statistically different from the measured U_EQ-5D_ (p-value<0.01) and U_VAS_ (p-value<0.01). In tobit regression analysis, factors independently predictive of U_EQ-5D_ included age and monthly household income. Patients aged ≥40 years old rated U_EQ-5D_ significantly lower than younger persons. Higher U_EQ-5D_ was significantly associated with higher monthly household income in a dose response fashion. The median U_EQ-5D_ was highest among patients who had been successfully treated for TB and lowest among multi-drug resistant TB (MDR-TB) patients who were on treatment.

**Conclusions:**

U_CAL_ of patients with two co-morbidities overestimated the measured utilities, warranting further research of how best to estimate utilities of patients with such conditions. TB and MDR-TB treatments impacted on patients' self perceived health status. This effect diminished after successful treatment.

## Introduction

Tuberculosis (TB) is a severe, often chronic, lung disease causing nearly nine million illnesses and more than one million deaths each year. [1] With appropriate treatment, approximately 90% of patients with active TB disease can be cured, even in patients with HIV infection. [2], [3] Despite the discovery of the first TB drug over 50 years ago, current treatment regimens for susceptible TB still require the use of a combination of potentially toxic antibiotics for a minimum of six months to ensure eradication. [4], [5] For multi-drug resistant TB (MDR-TB), available regimens are less potent (but more noxious), requiring longer treatment durations. [6], [7], [8] As such, patients' health-related quality of life (HRQL), i.e., valued aspects of life, may be diminished by side effects from medication, prolonged treatment duration, and in some cultures, social stigma attached to the disease. [9], [10], [11] TB disease itself may also have a negative impact on TB patients' self perceived health status. [12], [13]

Health systems in Thailand are increasingly cost effective. In an effort to respond better to patients' needs, healthcare providers are integrating services. [14], [15], [16] Economic and decision analyses are frequently conducted to inform resource allocation decisions. HRQL data are widely recognized as an important input in such exercises, particularly for chronic diseases. [17] HRQL can be derived using generic and specific instruments. [9] Generic instruments collect descriptive data and generate health utilities: preference-based, numeric representations of overall health that are the most commonly used measures for evaluating HRQL in economic analyses. [18] While health utilities, as input in cost-utility analyses, allow comparison between populations and across diseases, settings, and countries, information generated from specific instruments focus on problems associated with single disease states, patient groups, or areas of function and do not allow broad comparison. [9], [18], [19]

Our systematic review showed that data on formal assessment of HRQL in TB patients are rather sparse, particularly in the Thai setting. To date, there were only two studies conducted in Thai populations. [20], [21] The first study administered a generic instrument (Medical Outcomes Study 36-Item Short-From Health Survey) to measure HRQL among 84 pulmonary TB patients in Yunnan province of China and Southern Thailand. [20] Findings of this study, however, were published in Chinese, compromising dissemination of the results to non-Chinese speaking researchers. In 2008, Kittikraisak et al. conducted a prospective observational study to evaluate the impact of TB and HIV treatment on HRQL among 849 TB patients in Thailand. This study, however, focused on the patients who were HIV-infected and used a study specific questionnaire to collect HRQL data. [21] The investigators found impairment in physical and mental health when Thai HIV-infected patients studied were first diagnosed with TB. Additionally, completing TB treatment relieved some physical symptoms, but had little impact on mental health. Data, however, cannot be used for economic modeling purposes because neither generic nor specific instruments were employed to collect data. With increasing interest in identifying cost-effective interventions that are responsive to patients' needs, HRQL data collected using standardized instruments are urgently needed.

The main purpose of this study was to collect health utility data, using EuroQol 5D (EQ-5D) and EuroQol visual analogue scale (EQ-VAS) instruments from Thai TB patients and those cured or having completed treatment. The data were collected for use in our economic evaluation analysis of screening and diagnostic algorithms for pulmonary TB among HIV-infected patients in Thailand (to be published). In this study, we explored how socio-demographic characteristics and co-morbidity such as HIV infection affect TB patients' health utility and whether health utilities of patients with different medical conditions were different. Further, we examined concordance of health utilities measured using the two instruments. Lastly, we examined how health utility of patients with two morbidities calculated using multiplicative approach (U_CAL_) differed from the measured utilities.

## Methods

### Ethics statement

This study was approved by ethical review committees of Chiang Rai Regional Hospital and Bamrasnaradura Infectious Diseases Institute. Involvement of Centers for Disease Control and Prevention (CDC) investigators in this study was determined not to meet the definition of engagement in human subjects research per U.S. human subjects research regulations and additional review by the CDC institutional review board was not required. All participants had provided written informed consent.

### Study setting and population

From August to October 2009, we conducted a cross-sectional survey and recruited consecutive patients from respective clinics at Chiang Rai Regional Hospital and Bamrasnaradura Infectious Diseases Institute. These two hospitals were part of our multi-site population-based TB surveillance conducted in six provinces in Thailand and were chosen because they serve a high number of TB, HIV-infected TB, and HIV patients. [22] Eligible patients were those diagnosed with TB (including MDR-TB) and/or HIV ≥2 weeks before study enrollment to allow time for them to cope with the diagnosis(es), aged between 18–70 years old, who were able to communicate in Thai and did not require assistance from family members regarding communication, were not pregnant, and were not in the priesthood. Patients with HIV were recruited regardless of their anti-retroviral therapy (ART) status. After giving written informed consent, patients were enrolled and assigned into mutually exclusive groups according to their medical condition: 1) TB patients receiving TB treatment (TB_TX_), 2) MDR-TB patients receiving MDR-TB treatment (MDR_TX_), 3) patients who had been successfully treated for TB or MDR-TB according to World Health Organization definition and finished treatment for ≥6 months (_any_TB_C_), 4) HIV-infected patients at any stage who had not been diagnosed with TB (_any_HIV), 5) HIV-infected TB patients receiving TB treatment (TB_TX_/HIV), and 6) HIV-infected patients who had been successfully treated for TB or MDR-TB and finished treatment for ≥6 months (_any_TB_C_/HIV). Sample size that gave 80% power at the 0.05 level of significance (two-sided) was calculated as specified in the study protocol, with parameters estimated based on literature. An effect size of 7% for the difference between utilities of patients in different medical conditions was used in the calculation. Sample size of 32 patients per group was required for patients with TB only (groups 1–3). For HIV-infected patients regardless of TB status (groups 4–6), the required sample size was 49 per group. For groups 1, 2, and 5, we restricted enrollment to patients who were on TB or MDR-TB treatment for ≥2 weeks at enrollment, a long enough duration to experience side effect(s) from medications (if any). Each patient was compensated with 100 Thai Baht (∼3US$) for his/her time, except those in groups 3 and 6 who received additional 400 Baht for travel expenses because they were no longer visiting the hospitals for medical care.

### Data collection and instruments

At enrollment, trained study nurses administered: 1) structured questionnaire to collect socio-demographic characteristic data, 2) EQ-5D, and 3) EQ-VAS instruments (with permission to use from the developer [the EuroQol Group]) to collect patients' HRQL data. The two instruments are recommended by the Thai Health Technology Assessment Guidelines to be used to value health utility for economic purposes. [23] EQ-5D consists of five domains relating to: 1) mobility, 2) self-care, 3) usual activities, 4) pain/discomfort, and 5) anxiety/depression and was originally used as a self-administered questionnaire. [24] According to the developer, it can also be administered and used in postal surveys, in clinics, and face-to-face interviews. We conducted face to face interview and for each domain staff asked patients to assess their current health and respond with one of the following options: “no problem”, “moderate problem”, and “severe problem”. A color coded (green, yellow, and red) flip chart was used as a supplemental tool to enhance patients' understanding of the context. An EQ-5D health state of each respondent was recorded. For example, the “32211” health state implied the patient perceived a severe problem with mobility, a moderate problem with self-care and usual activities, but no pain/discomfort or anxiety/depression. Following EQ-5D administration, we asked patients to indicate their health status using EQ-VAS. The EQ-VAS is a standard visual analogue scale including a vertical line, 20 cm in length, anchored at “0” (death) at the bottom and “100” (full health) on top. We asked each patient to mark on the scale a rating of their health and well-being on the interview day. The instrument measures an individual's valuation of their current overall health status.

### Statistical analysis

The analysis was divided into four parts. First, we described socio-demographic and health characteristics. Second, we calculated EQ-5D utility value (U_EQ-5D_) by assuming that health utilities were additive and that the health utility of a person declined when his/her health deteriorated. [25] We transformed U_EQ-5D_ from the five-digit coded health states using an additive formula, including coefficients and a constant derived from the Thai utility score algorithm established from a recent national household survey in Thai general population. [26] Theoretically, U_EQ-5D_ ranges from 0.0 (death) to 1.0 (full health) value scale; scores less than zero representing states worse than death are possible. [27] We obtained EQ-VAS utilities (U_VAS_) by transforming them from a 0 to 100 directly to a 0 to 1 scale; scores lower than zero are not possible. Concordance between U_EQ-5D_ and U_VAS_ was estimated using “concord,” a user-written program for Stata by Steichen and Cox. [28], [29] Bland-Altman approach was used to examine agreement between the two scales used to measure utilities. [30]

Third, we fitted tobit regression models to examine associations between socio-demographic characteristics and U_EQ-5D_ and U_VAS_. We assessed whether there was any difference in health utility scores of patients in different medical conditions. Tobit regression models are designed to estimate linear relationships between variables when there is upper- or lower-censoring in the dependent variable. [31] We used an upper-censoring limit of one for analyses of U_EQ-5D_ and U_VAS_ (U_EQ-5D_ and U_VAS_ cannot exceed one), and a lower limit of zero for analysis of U_VAS_ (U_VAS_ cannot be lower than zero). The p-value of likelihood ratio chi-square was used as a guide to the model's goodness of fit. We used a two sided p-value of ≤0.05 to indicate statistical significance. However, to reduce the chances of a type I error, we employed a Bonferroni's adjustment when determining which groups of patients were different. [32] In this case, a p-value of ≤0.003 (0.05/15 comparisons) was considered significant. Lastly, we calculated health utilities of patients with two co-morbidities (e.g., TB and HIV) using multiplicative formula shown below and compared them with the actual data measured using EQ-5D and EQ-VAS. [33] All statistical analyses were conducted using Stata, version 10 (StataCorp LP, College Station, TX, USA).
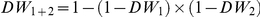


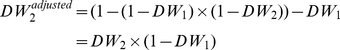
Where health utility (*U*) = 1−disability weight (*DW*)
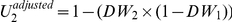
DW, disability weight; U, Health utility; subscript 1 stands for a more severe condition and subscript 2 stands for a milder condition.

## Results

### Socio-demographic characteristics of the population

During the enrollment period, 223 patients with TB and/or HIV were enrolled in the study. Of these, 222 were analyzed. We excluded MDR_TX_/HIV from the analysis because there was only one patient in this group. The analytic dataset included 32 TB_TX_, 11 MDR_TX_, 32 _any_TB_C_, 49 _any_HIV, 49 TB_TX_/HIV, and 49 _any_TB_C_/HIV. Of the 222 patients, 138 (62%) were male, 128 (58%) were married/cohabitating, and 172 (77%) finished either primary or high school.**[Table S1]** The median age at enrollment was 40 years (interquartile range [IQR], 35–47), 79 patients (36%) were laborers, and 203 (91%) were covered by health insurance. The median monthly household income was 6,000 Baht (IQR, 4,000–15,000). Among HIV-infected patients, 46 _any_HIV (94%), 26 TB_TX_/HIV (55%), and 38 _any_TB_C_/HIV (100%) reported currently receiving ART. These patients were diagnosed with and on treatment for HIV for a median of 36 months (IQR, 18–95) and 24 months (IQR, 2–51), respectively. TB patients were diagnosed with TB for a median of 3 months (IQR, 1–5). They were initiated treatment at the time of diagnosis, resulting in a median treatment duration of 3 months (IQR, 1–5). MDR-TB patients were diagnosed with the disease for a median of 9 months (IQR, 4–11); effective treatment was initiated relatively quickly after diagnosis. **Table 1** shows response of 222 patients to EQ-5D instrument stratified by medical conditions.

**Table 1:** Response of 222 Thai patients with various medical conditions to EuroQol 5D instrument, August to October 2009.

	All (n = 222)	TB_TX_ (n = 32)	MDR_TX_ (n = 11)	_any_TB_C_ (n = 32)	_any_HIV (n = 49)	TB_TX_/HIV (n = 49)	_any_TB_C_/HIV (n = 49)
	*n (%)*	*n (%)*	*n (%)*	*n (%)*	*n (%)*	*n (%)*	*n (%)*
Mobility							
No problem	172 (77)	21 (66)	5 (45)	28 (88)	41 (84)	32 (65)	45 (92)
Moderate problem	50 (23)	11 (34)	6 (55)	4 (12)	8 (16)	17 (35)	4 (8)
Severe problem	0 (0)	0 (0)	0 (0)	0 (0)	0 (0)	0 (0)	0 (0)
Self care							
No problem	210 (95)	26 (81)	9 (82)	32 (100)	48 (98)	47 (96)	48 (98)
Moderate problem	12 (5)	6 (19)	2 (18)	0 (0)	1 (2)	2 (4)	1 (2)
Severe problem	0 (0)	0 (0)	0 (0)	0 (0)	0 (0)	0 (0)	0 (0)
Usual activities							
No problem	161 (73)	22 (69)	3 (27)	29 (91)	40 (82)	26 (53)	41 (84)
Moderate problem	57 (26)	9 (28)	6 (55)	3 (9)	9 (18)	22 (45)	8 (16)
Severe problem	4 (1.8)	1 (3)	2 (18)	0 (0)	0 (0)	1 (2)	0 (0)
Pain/discomfort							
No problem	95 (43)	10 (31)	0 (0)	18 (56)	23 (47)	12 (24)	32 (65)
Moderate problem	122 (55)	21 (66)	9 (82)	14 (44)	25 (51)	36 (73)	17 (35)
Severe problem	5 (2)	1 (3)	2 (18)	0 (0)	1 (2)	1 (2)	0 (0)
Anxiety/depression							
No problem	125 (56)	15 (47)	3 (27)	25 (78)	27 (55)	23 (47)	32 (65)
Moderate problem	93 (42)	17 (53)	7 (64)	7 (22)	20 (41)	26 (53)	16 (33)
Severe problem	4 (2)	0 (0)	1 (9)	0 (0)	2 (4)	0 (0)	1 (2)

TB_TX_, TB patients receiving TB treatment; MDR_TX_, MDR-TB patients receiving MDR-TB treatment; _any_TB_C_, patients who had been successfully treated for TB or MDR-TB for ≥6 months; _any_HIV, HIV-infected patients at any stage; TB_TX_/HIV, HIV-infected TB patients receiving TB treatment; _any_TB_C_/HIV, HIV-infected patients who had been successfully treated for TB or MDR-TB for ≥6 months.

### Health utility measured using EQ-5D instrument

U_EQ-5D_ of the 222 patients ranged from −0.02 to 1.0 (median, 0.7; IQR, 0.6–1.0). One of eight MDR_TX_ (9%) perceived his overall health was worse than death (U_EQ-5D_, −0.02). This patient was diagnosed with and had been taking medication for MDR-TB for 26 months. By contrast, 7 TB_TX_ (22%), 16 _any_TB_C_ (50%), 17 _any_HIV (35%), 6 TB_TX_/HIV (12%), and 27 _any_TB_C_/HIV (55%) perceived they were in full health (U_EQ-5D_, 1.0). **Table 2** shows median U_EQ-5D_ of the 222 patients stratified by medical conditions. Overall, the median U_EQ-5D_ was highest among _any_TB_C_ (1.0; IQR, 0.7–1.0) and lowest among MDR_TX_ (0.5; IQR, 0.4–0.7).

**Table 2:** Health utilities of 222 Thai patients with various medical conditions measured using EuroQol 5D and EuroQol visual analogue score instruments, August to October 2009.

Patients	TB treatment	% receiving anti-retroviral therapy	N	Health utility by instrument			
				Median EQ-5D (IQR)	SD	Median EQ-VAS (IQR)	SD
TB	On TB treatment	Not applicable	32	0.69 (0.57–0.77)	0.22	0.80 (0.70–0.90)	0.15
MDR-TB	On MDR-TB treatment	Not applicable	11	0.51 (0.39–0.73)	0.21	0.60 (0.40–0.80)	0.25
TB or MDR-TB	Cured or completed treatment	Not applicable	32	0.88 (0.67–1.00)	0.17	0.85 (0.80–1.00)	0.15
HIV	Not applicable	94%	49	0.73 (0.63–1.00)	0.19	0.80 (0.70–0.90)	0.15
TB with HIV	On TB treatment	55%	49	0.67 (0.57–0.73)	0.16	0.70 (0.60–0.80)	0.16
TB or MDR-TB with HIV	Cured or completed treatment	100%	49	1.00 (0.69–1.00)	0.18	0.80 (0.70–0.90)	0.14
			222	0.73 (0.62–1.00)	0.21	0.80 (0.70–0.90)	0.17

TB, tuberculosis; MDR-TB, multi-drug resistant tuberculosis; EQ-5D, EuroQol 5D instrument; EQ-VAS, EuroQol visual analogue scale instrument; IQR, interquartile range; SD, standard deviation.

### Health utility measured using EQ-VAS instrument

The U_VAS_ ranged from 0.0 to 1.0 (median, 0.8; IQR, 0.7–0.9).**[** **Table 2** **]** The same MDR_TX_ with U_EQ-5D_ of −0.02 rated his health condition equivalent to death on EQ-VAS. Three TB_TX_ (9%), 10 _any_TB_C_ (31%), 10 _any_HIV (20%), 2 TB_TX_/HIV (4%), and 7 _any_TB_C_/HIV (14%) perceived they were in full health. The median U_VAS_ was relatively high in all groups of patients, except MDR_TX_ (0.6; IQR, 0.4–0.8) and TB_TX_/HIV (0.7; IQR, 0.6–0.8) whose median scores were slightly lower.

### Concordance between U_EQ-5D_ and U_VAS_

**Figure 1:**
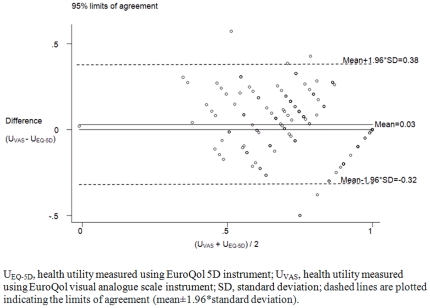
Bland-Altman plot showing the differences between health utilities measured using EuroQol 5D and EuroQol visual analogue scale instruments in relation to the mean of the two measurements in the 222 Thai patients, August to October 2009.

Concordance correlation coefficient between U_EQ-5D_ and U_VAS_ was 0.6 (95% confidence interval [CI], 0.5–0.7). Twenty-two patients (10%) rated equivalent health utilities on the two instruments. Eighty-six patients (39%) rated U_EQ-5D_ higher than U_VAS_; 114 patients (51%) rated U_EQ-5D_ lower than U_VAS_. Bland-Altman plot in **Figure 1** shows the differences between health utilities measured using EQ-5D and EQ-VAS instruments in relation to their means. Moderate agreement was observed. The 95% limits of agreement were shown at −0.32 and 0.38. The line of the average of the observed differences (average bias) was shown at 0.03.

### Predictors of health utility

In tobit regression analysis, factors independently predictive of U_EQ-5D_ included age and monthly household income.**[** **Table 3** **]** Patients aged ≥40 years old rated U_EQ-5D_ significantly lower than those aged <40 years old, adjusting for medical condition and monthly household income (estimate, −0.08; CI, −0.14 to −0.004). We found a dose response effect when examining impact of household income on U_EQ-5D_. Patients with monthly household income ≥5,000 Thai Baht rated U_EQ-5D_ significantly higher than patients with lower income, adjusting for medical condition and age.**[** **Table 3** **]** In the analysis of U_VAS_, we did not find any factor predictive of U_VAS_.

**Table 3:** Multivariate tobit regression analysis examining determinants of health utility of 222 Thai patients measured using EuroQol 5D instrument, August to October 2009.

	Estimates	95% confidence interval		p-value
		Lower	Upper	
Patient group				
TB_TX_	−0.24	−0.37	−0.10	<0.01
MDR_TX_	−0.41	−0.58	−0.24	<0.01
_any_TB_C_	ref	ref	ref	
_any_HIV	−0.13	−0.26	0.01	0.04
TB_TX_/HIV	−0.27	−0.39	−0.15	<0.01
_any_TB_C_/HIV	−0.01	−0.14	0.13	0.93
Age group (years)				
<40	ref	ref	ref	
≥40	−0.08	−0.15	−0.003	0.04
Monthly household income (Thai Baht)*				
<5,000	ref	ref	ref	
5,000–9,999	0.09	0.004	0.17	0.04
10,000–19,999	0.13	0.03	0.23	0.01
≥20,000	0.17	0.06	0.28	<0.01

TB, tuberculosis; MDR-TB, multi-drug resistant tuberculosis; TB_TX_, TB patients receiving TB treatment; MDR_TX_, MDR-TB patients receiving MDR-TB treatment; _any_TB_C_, patients who had been successfully treated for TB or MDR-TB for ≥6 months; _any_HIV, HIV-infected patients at any stage; TB_TX_/HIV, HIV-infected TB patients receiving TB treatment; _any_TB_C_/HIV, HIV-infected patients who had been successfully treated for TB or MDR-TB for ≥6 months; ref, referent group.

*32 Thai Baht = 1 US$.

### Difference in health utility

**Figure 2:**
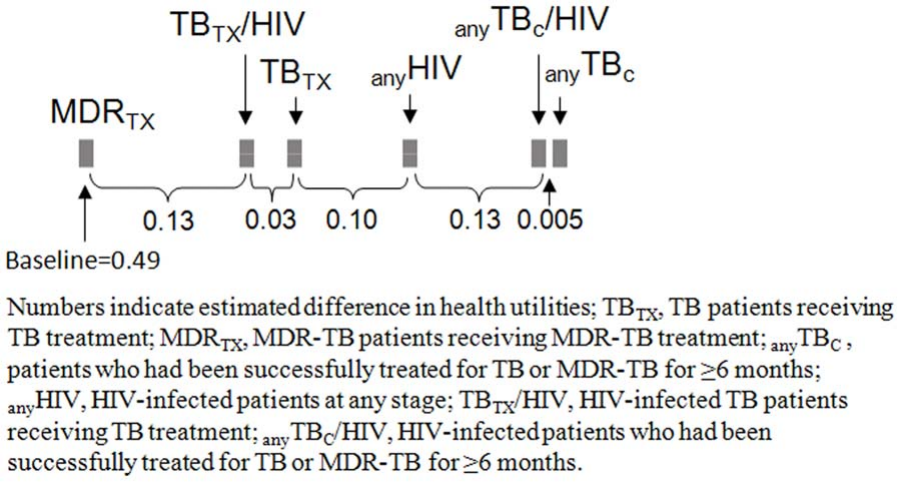
Schematic diagram showing estimated difference in health utilities of 222 Thai patients with various medical conditions adjusting for age and monthly household income and measured using EuroQol 5D instrument, August to October 2009.

We found that U_EQ-5D_ were highest in _any_TB_C_, followed by _any_TB_C_/HIV, _any_HIV, TB_TX_, TB_TX_/HIV, and MDR_TX_, adjusting for age and monthly household income. With Bonferroni's adjustment, patients could be divided, according to the fitted utilities, into three non-mutually exclusive groups: 1) _any_TB_C_, _any_TB_C_/HIV, _any_HIV; 2) _any_HIV, TB_TX_, TB_TX_/HIV; and 3) TB_TX_, TB_TX_/HIV, MDR_TX_. **Figure 2** shows estimated differences in health utilities of patients with various medical conditions, adjusting for age and monthly household income. In the analysis of U_VAS_, we found that U_VAS_ of patients in different groups ranked in the same order as that seen in the analysis of U_EQ-5D_. However, because the model fitted poorly we did not further examine if and how each group of patients differed from one another.

### U_CAL_ versus the actual data

The median health utility of 49 TB_TX_/HIV was relatively high (0.7 for both U_EQ-5D_ and U_VAS_). U_CAL_ for patients with TB and HIV co-infection (0.8) was statistically different from the measured U_EQ-5D_ (p<0.01), and U_VAS_ (p<0.01). Of the 49 TB_TX_/HIV, 43 (88%) rated U_EQ-5D_ lower than U_CAL_. Six (12%) rated U_EQ-5D_ higher than U_CAL_. None of TB_TX_/HIV rated U_EQ-5D_ equal to U_CAL_. Likewise, 31 patients (63%) rated U_VAS_ lower than U_CAL_. Nine (18%) rated U_VAS_ higher than U_CAL_. Nine patients (18%) rated U_VAS_ equal to U_CAL_.

## Discussion

In this study, we found that the Thai language EQ-5D and EQ-VAS instruments could be used for measuring and evaluating health utility in a selected group of TB and HIV patients. Further, patients' age and monthly household income were found to be determinants of U_EQ-5D_. TB and MDR-TB treatment may impact health utilities of patients receiving such treatment. This effect diminished after successful treatment of the disease. Health utilities of patients with HIV and TB calculated using multiplicative model for two co-morbidities overestimated the directly measured utilities.

To our knowledge, this is the first study that elicited health utility in HIV-infected TB patients and compared health utilities between HIV-infected and HIV-uninfected TB patients. Our study is also the first study demonstrating feasibility of the Thai language EQ-5D and EQ-VAS instruments in measuring health utility in a Thai TB population regardless of HIV-infection. The English versions of the instruments were recommended for use in all groups of patients and the Thai versions have recently been ratified by the EuroQol Group's Translation Committee. [34], [35] Both instruments could identify differences in health utilities among patients with different medical conditions. In this study, more than half were elderly or adults with co-morbidity who had finished basic schooling. All successfully completed the study task using the EQ-5D and EQ-VAS. We believe that this would not be possible with the original English language self-administered instruments. It should be noted that assistance from study personnel was only to read questions on instruments to participants who had difficulty reading. Color-coded supplemental tool was used to help participants who had trouble remembering answer choices. Further, a study by Puhan et al has documented that administration formats do not have a meaningful effect on repeated measurements of patient-reported HRQL outcomes. [36] While our study was conducted among a sample of TB patients, the socio-demographic and health characteristics of our population were similar to those of the population-based TB surveillance network in Thailand. This suggests our findings may be generalisable to the wider Thai TB population. [22], [37]

Consistent with previously studies, higher HRQL, the U_EQ-5D_ in our study, was correlated with younger age and higher household income, likely because of better prognosis. [12], [38], [39], [40] Yet, we did not find significant associations between sex, education, health insurance coverage, and HRQL as found in other studies or any predictor for U_VAS_. [39], [41] This may be due to different characteristics of the populations studied. Our population was out-patients receiving services at hospitals and more than 90% were covered by health insurance. In contrast, those in Duyan's study were hospitalized TB patients with low levels of education and no social insurance coverage. [39] Additionally, those in both Duyan's and Nyamathi's studies reported insufficient housing conditions. [39], [41]

The U_EQ-5D_ and U_VAS_ obtained from this study were in line with other studies which suggested that impaired health utility occurred during TB and MDR-TB treatments. [21], [38], [42] Nearly half of our TB patients were still in the intensive phase of TB treatment, making them more prone to disutility. Moreover, 63% of MDR-TB had been on treatment for more than six months with one patient being on treatment for more than two years. This finding together with those from our previous study suggests that provision of a more holistic approach to medical care not limited only to HIV and TB treatment may be beneficial to the patients. [21] Interventions focusing on symptom management and coordination of care may help relieve symptoms and improve patients' ability to tolerate medical treatment as well as help them gain the strength to carry on with daily life. In this study, we also found that improved health utility after TB treatment was more pronounced in HIV-infected patients than those uninfected in nearly all domains. This is likely due to relief of some TB symptoms and adverse events from HIV and TB drug interactions. [21], [43] Our study did not measure markers of disease progression (e.g., CD4+ T-lymphocyte) among those HIV-infected. Nonetheless, studies have reported that HIV-infected patients with or without AIDS appeared to have similar levels of HRQL in the era of highly active ART. This could likely be explained by the effectiveness of medication in reversing the progression of disease in individuals with AIDS and accommodation to the stress of living with the disease. [44], [45] In fact, over 80% of HIV-infected patients in our study were receiving ART and were among those whose U_EQ-5D_ were highest. This finding implies that ART delivery in the public sector of Thai healthcare system may have an impact not only on patients' survival as has been found in other studies, but also HRQL and ability to function in society. [46], [47], [48]

It is noteworthy that health utilities of persons with two co-morbidities calculated using multiplicative model were overestimated compared to those measured directly using EQ-5D and EQ-VAS. Because co-morbidities are common, this finding warrants further research of how best to estimate utilities of patients with such conditions.

There are a number of limitations in our study. First, enrollment of patients was not done in a random or systematic manner due to operational constraints. As mentioned, socio-demographic and health characteristics of our patients were similar to those in a multi-site population-based TB surveillance system, suggesting interviewed patients may be broadly representative. Second, there was only one MDR_TX_/HIV enrolled in our study; this patient was subsequently excluded from the analysis because of small sample size. This implies the rarity of this sub-population in Thailand. HRQL in this particular group remains an open question that needs to be addressed by future research in settings where MDR_TX_/HIV is more prevalent. Further, the required sample size for MDR_TX_ was not met, prompting caution when interpreting data of this particular group of patients. Third, we did not further stratify patients based on sputum smear microscopy results because of the restriction to enrol only patients who had received TB treatment for ≥2 weeks. Some of these patients were expected to have a conversion by the interview time. In India, Dhingra and Rajpal have documented difference in HRQL between smear positive and negative TB patients using a TB-specific instrument. [49] We were unable to investigate if this difference existed in our study. Fourth, screening for active TB among our HIV-infected patients may not have been optimal. It is possible that some patients may have had undiagnosed TB, resulting in misclassification. However, patients in our study were routinely asked if they had coughed along with other symptoms. This information was passed to attending physicians. Therefore, we believe that number of undiagnosed TB should be small. Lastly, as for other HRQL instruments, the EQ-5D and EQ-VAS reflect patients' opinions. Different individuals assign different values to the same health state, and consequently vary in their preferences. Further, as pointed out by Aghakhani et al, the overall responses to the EQ-5D instrument (which has three possible answers) may be forced to the mid-range category because few patients endorse the ‘severe’ value and some limitation is often present. This possibly results in diverting responses away from the ‘no limitation’ option. [50] The EQ-5D has been critiqued as less sensitive than disease-specific measurements resulting in possible overestimation of patients' HRQL. Nonetheless, because one of the study goals was to identify utility values that could be used for economic modelling in the future, the ability to compare across diseases outweighed the sensitivity concerns.

In resource-limited settings, economic analysis is increasingly carried out to inform practice guidelines, funding decisions, and research initiatives. Utility data collected from our study may be incorporated into cost-effectiveness and cost-utility analyses. These in turn allow TB control strategies to be compared more directly with other public health interventions, with respect to both costs and consequences and whether the interventions are of benefit in relation to HRQL. Our findings also suggest that the EQ-5D and EQ-VAS have discriminative power and are responsive to clinically important changes related to TB treatment.

## Supporting Information

Table S1 Socio-demographic and health characteristics of 222 Thai patients with various medical conditions, August to October 2009.^*^
(DOCX)
