# Supplementary material for: Health Related Quality of Life among Patients with Tuberculosis and HIV in Thailand
Source: PLoS One. 2012 Jan 11;7(1):e29775. doi: 10.1371/journal.pone.0029775 (PMC3256183; doi:10.1371/journal.pone.0029775)
Supplement: Table S1 — Socio-demographic and health characteristics of 222 Thai patients with various medical conditions, August to October 2009.* (DOCX) [file pone.0029775.s001.docx]

|  | All (n=222) | TB_TX_ (n=32) | MDR_TX_ (n=11) | _any_TB_C_ (n=32) | _any_HIV (n=49) | TB_TX_/HIV (n=49) | _any_TB_C_/HIV (n=49) |
| --- | --- | --- | --- | --- | --- | --- | --- |
|  | *n (%)* | *n (%)* | *n (%)* | *n (%)* | *n (%)* | *n (%)* | *n (%)* |
| Sex |  |  |  |  |  |  |  |
| Male | 138 (62) | 18 (56) | 6 (55) | 25 (78) | 27 (55) | 32 (65) | 30 (61) |
| Female | 84 (38) | 14 (44) | 5 (45) | 7 (22) | 22 (45) | 17 (35) | 19 (39) |
| Marital status |  |  |  |  |  |  |  |
| Single | 40 (18) | 7 (22) | 2 (18) | 5 (16) | 7 (14) | 9 (18) | 10 (20) |
| Married/cohabitating | 128 (58) | 20 (63) | 7 (64) | 25 (78) | 27 (55) | 27 (55) | 22 (45) |
| Widow | 32 (14) | 3 (9) | 1 (9) | 0 (0) | 10 (20) | 7 (14) | 11 (22) |
| Divorced/separated | 22 (10) | 2 (6) | 1 (9) | 2 (6) | 5 (10) | 6 (12) | 6 (12) |
| Education |  |  |  |  |  |  |  |
| Never attended school | 17 (8) | 6 (19) | 1 (9) | 3 (10) | 4 (8) | 2 (4) | 1 (2) |
| Primary school | 102 (46) | 12 (37) | 7 (64) | 16 (50) | 23 (47) | 23 (47) | 21 (43) |
| High school/vocational certificate | 70 (32) | 10 (31) | 1 (9) | 7 (22) | 13 (27) | 17 (35) | 22 (45) |
| Diploma/high vocational certificate | 11 (5) | 0 (0) | 1 (9) | 2 (6) | 2 (4) | 2 (4) | 4 (8) |
| Bachelor or equivalent/post graduate degree | 22 (10) | 4 (13) | 1 (9) | 4 (13) | 7 (14) | 5 (10) | 1 (2) |
| Occupation |  |  |  |  |  |  |  |
| Agriculture/fisheries | 35 (16) | 5 (16) | 0 (0) | 13 (41) | 5 (10) | 4 (8) | 8 (16) |
| Business owner | 35 (16) | 2 (6) | 2 (19) | 4 (13) | 9 (18) | 11 (22) | 7 (14) |
| Laborer | 79 (36) | 9 (28) | 4 (37) | 11 (34) | 19 (39) | 17 (35) | 19 (39) |
| Private company employee | 15 (7) | 5 (16) | 0 (0) | 1 (3) | 3 (6) | 3 (6) | 3 (6) |
| Government/state enterprise officer | 17 (8) | 3 (9) | 1 (9) | 2 (6) | 4 (8) | 3 (6) | 4 (8) |
| Housekeeper | 17 (8) | 3 (9) | 2 (18) | 1 (3) | 7 (14) | 1 (2) | 3 (6) |
| Student/retiree/unemployed | 24 (11) | 5 (16) | 2 (18) | 0 (0) | 2 (4) | 10 (20) | 5 (10) |
| Health insurance |  |  |  |  |  |  |  |
| Universal Coverage Scheme | 146 (66) | 17 (53) | 7 (64) | 22 (69) | 35 (71) | 30 (61) | 35 (71) |
| Social Security Scheme | 37 (17) | 7 (22) | 2 (18) | 6 (19) | 4 (8) | 10 (20) | 8 (16) |
| Civil Servant Medical Benefit Scheme | 20 (9) | 4 (12) | 2 (18) | 3 (9) | 4 (8) | 4 (8) | 3 (6) |
| Out of pocket | 19 (9) | 4 (12) | 0 (0) | 1 (3) | 6 (12) | 5 (10) | 3 (6) |
| Age (years) | 40 (35-47) | 44.5 (36-51) | 50 (35-66) | 47.5 (38.5-56.5) | 41 (36-47) | 37 (32-41) | 38 (35-44) |
| Household income (Thai Baht)^†^ | 6,000 (4,000-15,000) | 6,000 (5,000-15,500) | 3,500 (1,800-7,800) | 5,750 (4,000-13,500) | 7,000 (5,000-17,000) | 7,500 (4,000-12,000) | 8,000 (4,000-12,000) |
| Duration from TB or MDR-TB diagnosis (months) | 5 (2-15) | 2.5 (1-5) | 9 (4-11) | 24.5 (16.5-34.5) | - | 24 (12-34) | 3 (1-6) |
| Duration from TB or MDR-TB treatment initiation (months) | 3 (1-6) | 2.5 (1-5) | 9 (4-11) | - | - | 3 (1-5) | - |
| Duration from HIV diagnosis (months) | 36 (18-95) | - | - | - | 84 (35-124) | 14 (5-52) | 36 (27-60) |
| Duration from anti-retroviral initiation (months) | 24 (2-51) | - | - | - | 51 (15-82) | 2 (0-8) | 28 (23-36) |

TB, tuberculosis; MDR-TB, multi-drug resistant tuberculosis; TB_TX_, TB patients receiving TB treatment; MDR_TX_, MDR-TB patients receiving MDR-TB treatment; _any_TB_C_ , patients who had been successfully treated for TB or MDR-TB for ≥6 months; _any_HIV, HIV-infected patients at any stage; TB_TX_/HIV, HIV-infected TB patients receiving TB treatment; _any_TB_C_/HIV, HIV-infected patients who had been successfully treated for TB or MDR-TB for ≥6 months; IQR, interquartile range.

^*^One HIV-infected patient who was on MDR-TB treatment was excluded due to small sample size.

^†^32 Thai Baht=1 US$.
